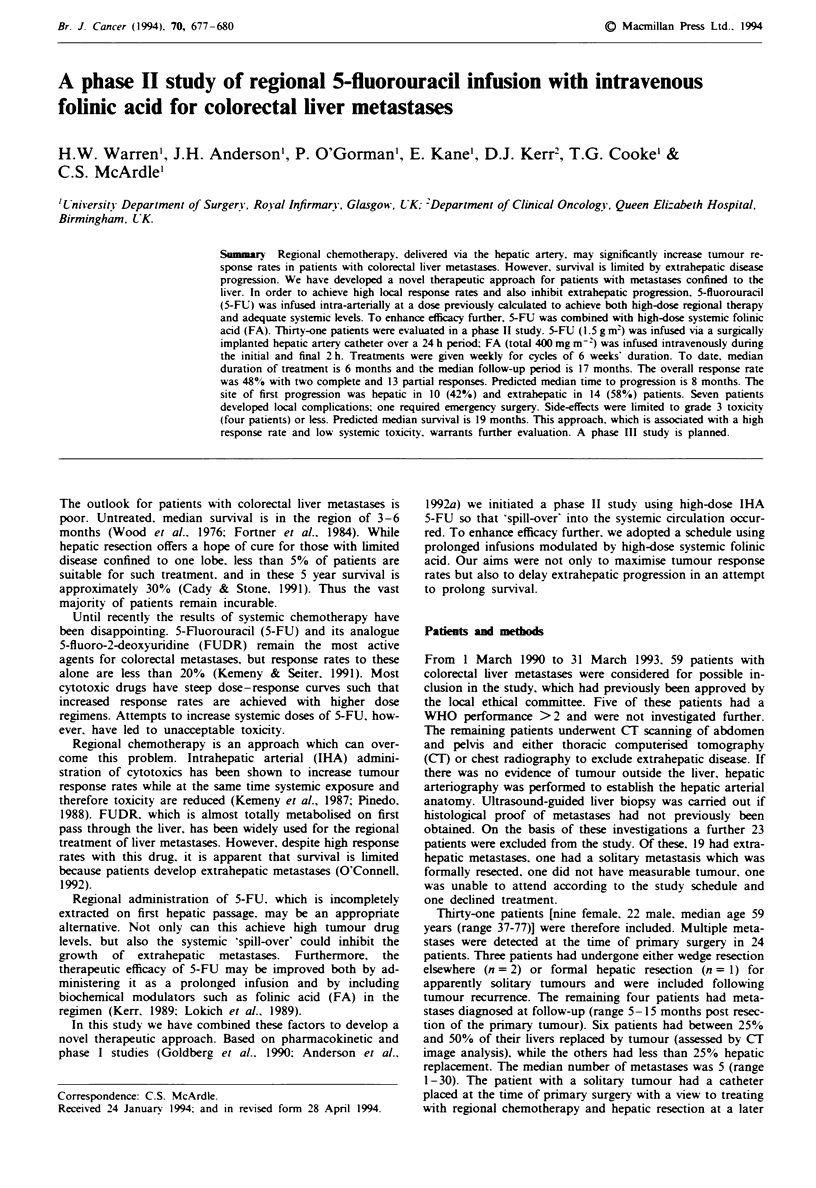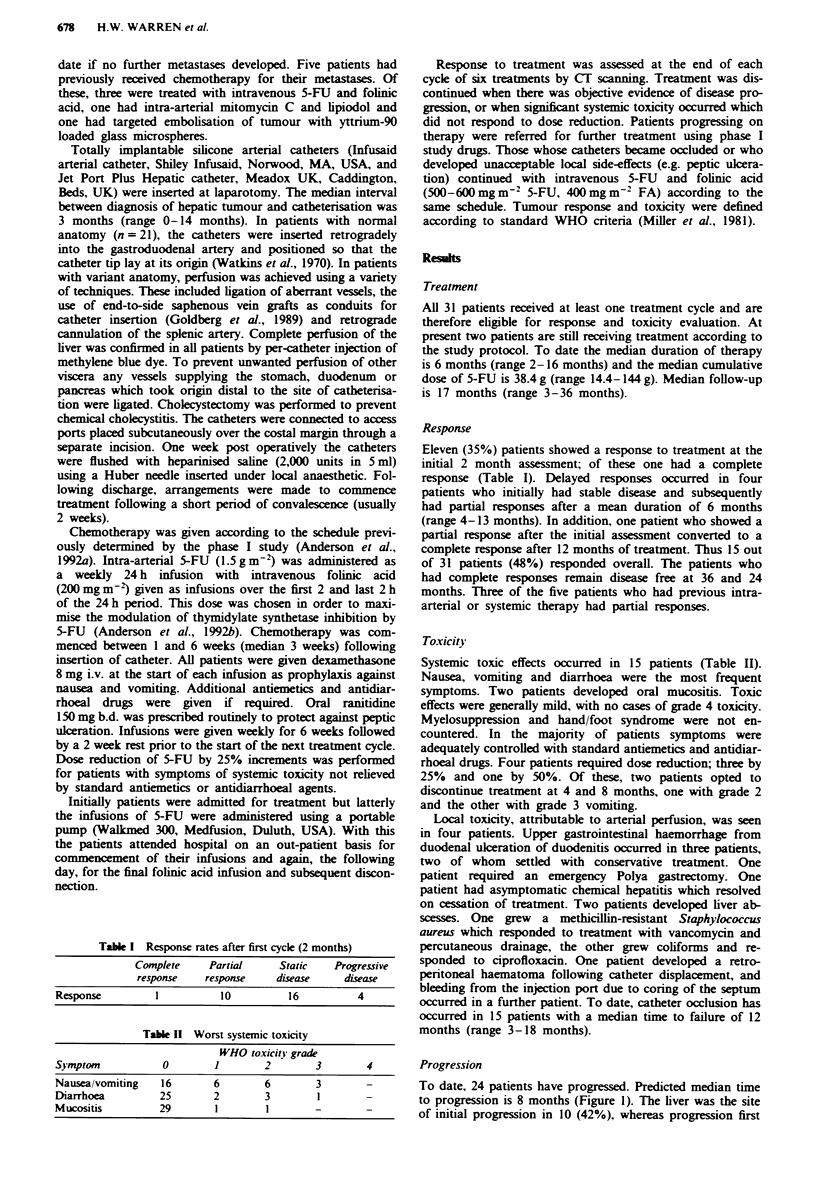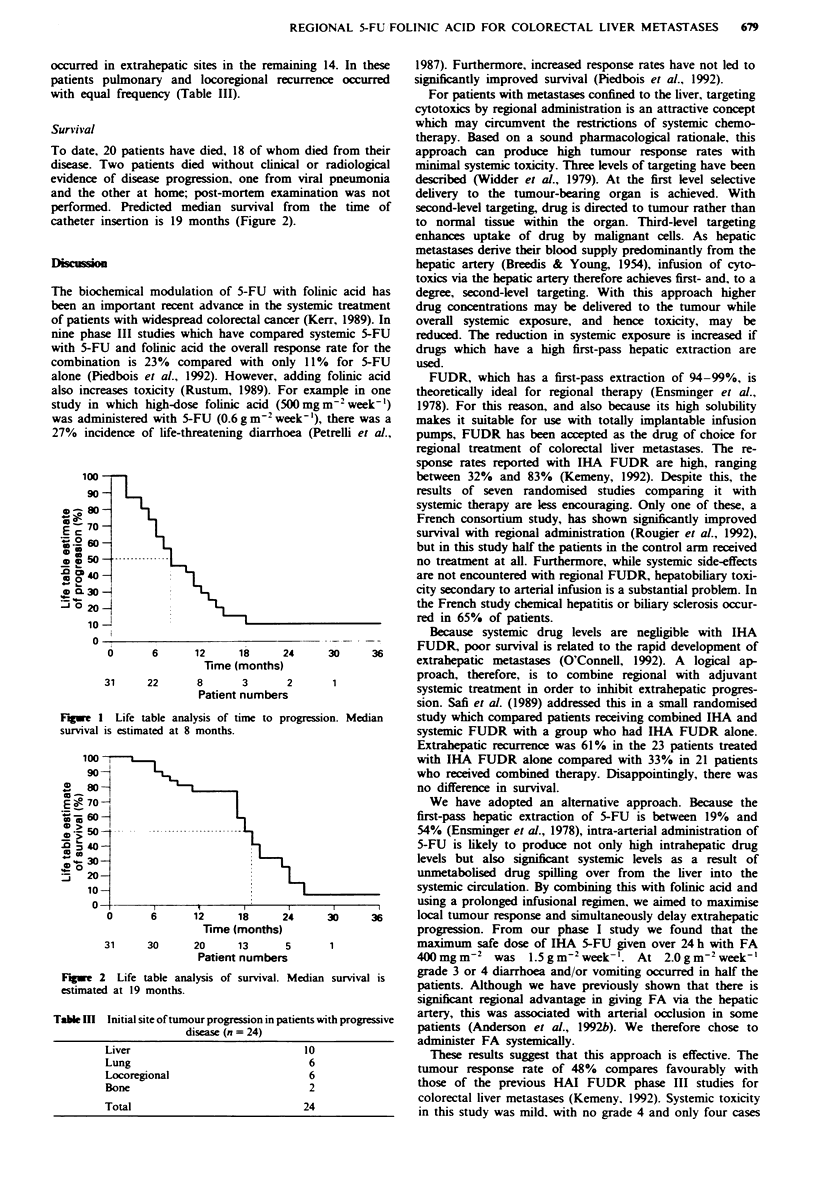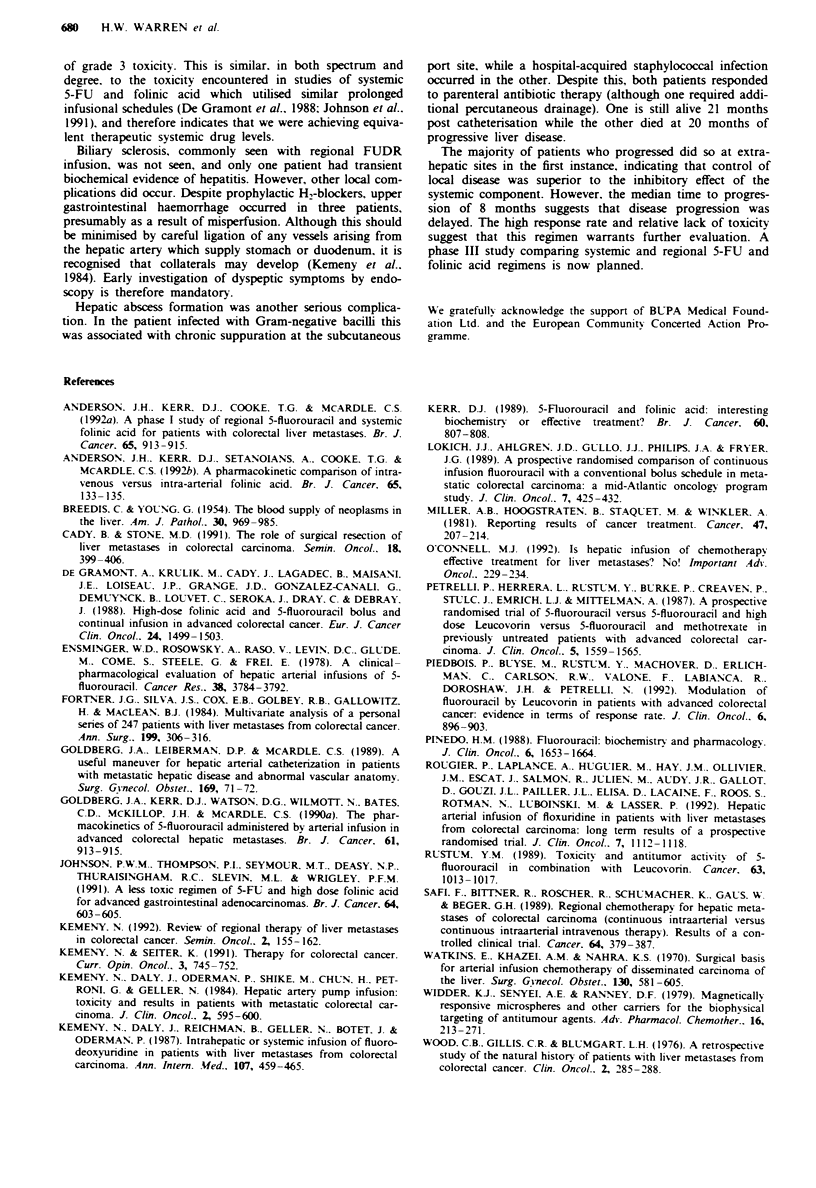# A phase II study of regional 5-fluorouracil infusion with intravenous folinic acid for colorectal liver metastases.

**DOI:** 10.1038/bjc.1994.371

**Published:** 1994-10

**Authors:** H. W. Warren, J. H. Anderson, P. O'Gorman, E. Kane, D. J. Kerr, T. G. Cooke, C. S. McArdle

**Affiliations:** University Department of Surgery, Royal Infirmary, Glasgow, UK.

## Abstract

Regional chemotherapy, delivered via the hepatic artery, may significantly increase tumour response rates in patients with colorectal liver metastases. However, survival is limited by extrahepatic disease progression. We have developed a novel therapeutic approach for patients with metastases confined to the liver. In order to achieve high local response rates and also inhibit extrahepatic progression, 5-fluorouracil (5-FU) was infused intra-arterially at a dose previously calculated to achieve both high-dose regional therapy and adequate systemic levels. To enhance efficacy further, 5-FU was combined with high-dose systemic folinic acid (FA). Thirty-one patients were evaluated in a phase II study. 5-FU (1.5 g m2) was infused via a surgically implanted hepatic artery catheter over a 24 h period; FA (total 400 mg m-2) was infused intravenously during the initial and final 2 h. Treatments were given weekly for cycles of 6 weeks' duration. To date, median duration of treatment is 6 months and the median follow-up period is 17 months. The overall response rate was 48% with two complete and 13 partial responses. Predicted median time to progression is 8 months. The site of first progression was hepatic in 10 (42%) and extrahepatic in 14 (58%) patients. Seven patients developed local complications; one required emergency surgery. Side-effects were limited to grade 3 toxicity (four patients) or less. Predicted median survival is 19 months. This approach, which is associated with a high response rate and low systemic toxicity, warrants further evaluation. A phase III study is planned.


Br. J. Cancer (1994), 70, 677-680 © Macmillan Press Ltd., 1994

### A phase II study of regional 5-fluorouracil infusion with intravenous folinic acid for colorectal liver metastases

H.W. Warren[1], J.H. Anderson[1], P. O'Gorman[1], E. Kane[1], D.J. Kerr[2], T.G. Cooke[1] & C.S. McArdle[1]

[1]*University Department of Surgery, Royal Infirmary, Glasgow, UK;* [2]*Department of Clinical Oncology, Queen Elizabeth Hospital, Birmingham, UK.*

**Summary** Regional chemotherapy, delivered via the hepatic artery, may significantly increase tumour response rates in patients with colorectal liver metastases. However, survival is limited by extrahepatic disease progression. We have developed a novel therapeutic approach for patients with metastases confined to the liver. In order to achieve high local response rates and also inhibit extrahepatic progression, 5-fluorouracil (5-FU) was infused intra-arterially at a dose previously calculated to achieve both high-dose regional therapy and adequate systemic levels. To enhance efficacy further, 5-FU was combined with high-dose systemic folinic acid (FA). Thirty-one patients were evaluated in a phase II study. 5-FU (1.5 g $m^2$) was infused via a surgically implanted hepatic artery catheter over a 24 h period; FA (total 400 mg $m^{-2}$) was infused intravenously during the initial and final 2 h. Treatments were given weekly for cycles of 6 weeks' duration. To date, median duration of treatment is 6 months and the median follow-up period is 17 months. The overall response rate was 48% with two complete and 13 partial responses. Predicted median time to progression is 8 months. The site of first progression was hepatic in 10 (42%) and extrahepatic in 14 (58%) patients. Seven patients developed local complications; one required emergency surgery. Side-effects were limited to grade 3 toxicity (four patients) or less. Predicted median survival is 19 months. This approach, which is associated with a high response rate and low systemic toxicity, warrants further evaluation. A phase III study is planned.

The outlook for patients with colorectal liver metastases is poor. Untreated, median survival is in the region of 3-6 months (Wood *et al.*, 1976; Fortner *et al.*, 1984). While hepatic resection offers a hope of cure for those with limited disease confined to one lobe, less than 5% of patients are suitable for such treatment, and in these 5 year survival is approximately 30% (Cady & Stone, 1991). Thus the vast majority of patients remain incurable.

Until recently the results of systemic chemotherapy have been disappointing. 5-Fluorouracil (5-FU) and its analogue 5-fluoro-2-deoxyuridine (FUDR) remain the most active agents for colorectal metastases, but response rates to these alone are less than 20% (Kemeny & Seiter, 1991). Most cytotoxic drugs have steep dose-response curves such that increased response rates are achieved with higher dose regimens. Attempts to increase systemic doses of 5-FU, however, have led to unacceptable toxicity.

Regional chemotherapy is an approach which can overcome this problem. Intrahepatic arterial (IHA) administration of cytotoxics has been shown to increase tumour response rates while at the same time systemic exposure and therefore toxicity are reduced (Kemeny *et al.*, 1987; Pinedo, 1988). FUDR, which is almost totally metabolised on first pass through the liver, has been widely used for the regional treatment of liver metastases. However, despite high response rates with this drug, it is apparent that survival is limited because patients develop extrahepatic metastases (O'Connell, 1992).

Regional administration of 5-FU, which is incompletely extracted on first hepatic passage, may be an appropriate alternative. Not only can this achieve high tumour drug levels, but also the systemic 'spill-over' could inhibit the growth of extrahepatic metastases. Furthermore, the therapeutic efficacy of 5-FU may be improved both by administering it as a prolonged infusion and by including biochemical modulators such as folinic acid (FA) in the regimen (Kerr, 1989; Lokich *et al.*, 1989).

In this study we have combined these factors to develop a novel therapeutic approach. Based on pharmacokinetic and phase I studies (Goldberg *et al.*, 1990; Anderson *et al.*, 1992*a*) we initiated a phase II study using high-dose IHA 5-FU so that 'spill-over' into the systemic circulation occurred. To enhance efficacy further, we adopted a schedule using prolonged infusions modulated by high-dose systemic folinic acid. Our aims were not only to maximise tumour response rates but also to delay extrahepatic progression in an attempt to prolong survival.

### Patients and methods

From 1 March 1990 to 31 March 1993, 59 patients with colorectal liver metastases were considered for possible inclusion in the study, which had previously been approved by the local ethical committee. Five of these patients had a WHO performance >2 and were not investigated further. The remaining patients underwent CT scanning of abdomen and pelvis and either thoracic computerised tomography (CT) or chest radiography to exclude extrahepatic disease. If there was no evidence of tumour outside the liver, hepatic arteriography was performed to establish the hepatic arterial anatomy. Ultrasound-guided liver biopsy was carried out if histological proof of metastases had not previously been obtained. On the basis of these investigations a further 23 patients were excluded from the study. Of these, 19 had extrahepatic metastases, one had a solitary metastasis which was formally resected, one did not have measurable tumour, one was unable to attend according to the study schedule and one declined treatment.

Thirty-one patients [nine female, 22 male, median age 59 years (range 37-77)] were therefore included. Multiple metastases were detected at the time of primary surgery in 24 patients. Three patients had undergone either wedge resection elsewhere ($n = 2$) or formal hepatic resection ($n = 1$) for apparently solitary tumours and were included following tumour recurrence. The remaining four patients had metastases diagnosed at follow-up (range 5-15 months post resection of the primary tumour). Six patients had between 25% and 50% of their livers replaced by tumour (assessed by CT image analysis), while the others had less than 25% hepatic replacement. The median number of metastases was 5 (range 1-30). The patient with a solitary tumour had a catheter placed at the time of primary surgery with a view to treating with regional chemotherapy and hepatic resection at a later

Correspondence: C.S. McArdle.
Received 24 January 1994; and in revised form 28 April 1994.

date if no further metastases developed. Five patients had previously received chemotherapy for their metastases. Of these, three were treated with intravenous 5-FU and folinic acid, one had intra-arterial mitomycin C and lipiodol and one had targeted embolisation of tumour with yttrium-90 loaded glass microspheres.

Totally implantable silicone arterial catheters (Infusaid arterial catheter, Shiley Infusaid, Norwood, MA, USA, and Jet Port Plus Hepatic catheter, Meadox UK, Caddington, Beds, UK) were inserted at laparotomy. The median interval between diagnosis of hepatic tumour and catheterisation was 3 months (range 0–14 months). In patients with normal anatomy ($n = 21$), the catheters were inserted retrogradely into the gastroduodenal artery and positioned so that the catheter tip lay at its origin (Watkins *et al.*, 1970). In patients with variant anatomy, perfusion was achieved using a variety of techniques. These included ligation of aberrant vessels, the use of end-to-side saphenous vein grafts as conduits for catheter insertion (Goldberg *et al.*, 1989) and retrograde cannulation of the splenic artery. Complete perfusion of the liver was confirmed in all patients by per-catheter injection of methylene blue dye. To prevent unwanted perfusion of other viscera any vessels supplying the stomach, duodenum or pancreas which took origin distal to the site of catheterisation were ligated. Cholecystectomy was performed to prevent chemical cholecystitis. The catheters were connected to access ports placed subcutaneously over the costal margin through a separate incision. One week post operatively the catheters were flushed with heparinised saline (2,000 units in 5 ml) using a Huber needle inserted under local anaesthetic. Following discharge, arrangements were made to commence treatment following a short period of convalescence (usually 2 weeks).

Chemotherapy was given according to the schedule previously determined by the phase I study (Anderson *et al.*, 1992*a*). Intra-arterial 5-FU (1.5 g m$^{-2}$) was administered as a weekly 24 h infusion with intravenous folinic acid (200 mg m$^{-2}$) given as infusions over the first 2 and last 2 h of the 24 h period. This dose was chosen in order to maximise the modulation of thymidylate synthetase inhibition by 5-FU (Anderson *et al.*, 1992*b*). Chemotherapy was commenced between 1 and 6 weeks (median 3 weeks) following insertion of catheter. All patients were given dexamethasone 8 mg i.v. at the start of each infusion as prophylaxis against nausea and vomiting. Additional antiemetics and antidiarrhoeal drugs were given if required. Oral ranitidine 150 mg b.d. was prescribed routinely to protect against peptic ulceration. Infusions were given weekly for 6 weeks followed by a 2 week rest prior to the start of the next treatment cycle. Dose reduction of 5-FU by 25% increments was performed for patients with symptoms of systemic toxicity not relieved by standard antiemetics or antidiarrhoeal agents.

Initially patients were admitted for treatment but latterly the infusions of 5-FU were administered using a portable pump (Walkmed 300, Medfusion, Duluth, USA). With this the patients attended hospital on an out-patient basis for commencement of their infusions and again, the following day, for the final folinic acid infusion and subsequent disconnection.

**Table I** Response rates after first cycle (2 months)

| | *Complete response* | *Partial response* | *Static disease* | *Progressive disease* |
|---|---|---|---|---|
| Response | 1 | 10 | 16 | 4 |

**Table II** Worst systemic toxicity

| | *WHO toxicity grade* | | | | |
|---|---|---|---|---|---|
| *Symptom* | *0* | *1* | *2* | *3* | *4* |
| Nausea/vomiting | 16 | 6 | 6 | 3 | – |
| Diarrhoea | 25 | 2 | 3 | 1 | – |
| Mucositis | 29 | 1 | 1 | – | – |

Response to treatment was assessed at the end of each cycle of six treatments by CT scanning. Treatment was discontinued when there was objective evidence of disease progression, or when significant systemic toxicity occurred which did not respond to dose reduction. Patients progressing on therapy were referred for further treatment using phase I study drugs. Those whose catheters became occluded or who developed unacceptable local side-effects (e.g. peptic ulceration) continued with intravenous 5-FU and folinic acid (500–600 mg m$^{-2}$ 5-FU, 400 mg m$^{-2}$ FA) according to the same schedule. Tumour response and toxicity were defined according to standard WHO criteria (Miller *et al.*, 1981).

### Results

### *Treatment*

All 31 patients received at least one treatment cycle and are therefore eligible for response and toxicity evaluation. At present two patients are still receiving treatment according to the study protocol. To date the median duration of therapy is 6 months (range 2–16 months) and the median cumulative dose of 5-FU is 38.4 g (range 14.4–144 g). Median follow-up is 17 months (range 3–36 months).

### *Response*

Eleven (35%) patients showed a response to treatment at the initial 2 month assessment; of these one had a complete response (Table I). Delayed responses occurred in four patients who initially had stable disease and subsequently had partial responses after a mean duration of 6 months (range 4–13 months). In addition, one patient who showed a partial response after the initial assessment converted to a complete response after 12 months of treatment. Thus 15 out of 31 patients (48%) responded overall. The patients who had complete responses remain disease free at 36 and 24 months. Three of the five patients who had previous intra-arterial or systemic therapy had partial responses.

### *Toxicity*

Systemic toxic effects occurred in 15 patients (Table II). Nausea, vomiting and diarrhoea were the most frequent symptoms. Two patients developed oral mucositis. Toxic effects were generally mild, with no cases of grade 4 toxicity. Myelosuppression and hand/foot syndrome were not encountered. In the majority of patients symptoms were adequately controlled with standard antiemetics and antidiarrhoeal drugs. Four patients required dose reduction; three by 25% and one by 50%. Of these, two patients opted to discontinue treatment at 4 and 8 months, one with grade 2 and the other with grade 3 vomiting.

Local toxicity, attributable to arterial perfusion, was seen in four patients. Upper gastrointestinal haemorrhage from duodenal ulceration of duodenitis occurred in three patients, two of whom settled with conservative treatment. One patient required an emergency Polya gastrectomy. One patient had asymptomatic chemical hepatitis which resolved on cessation of treatment. Two patients developed liver abscesses. One grew a methicillin-resistant *Staphylococcus aureus* which responded to treatment with vancomycin and percutaneous drainage, the other grew coliforms and responded to ciprofloxacin. One patient developed a retroperitoneal haematoma following catheter displacement, and bleeding from the injection port due to coring of the septum occurred in a further patient. To date, catheter occlusion has occurred in 15 patients with a median time to failure of 12 months (range 3–18 months).

### *Progression*

To date, 24 patients have progressed. Predicted median time to progression is 8 months (Figure 1). The liver was the site of initial progression in 10 (42%), whereas progression first occurred in extrahepatic sites in the remaining 14. In these patients pulmonary and locoregional recurrence occurred with equal frequency (Table III).

*Survival*

To date, 20 patients have died, 18 of whom died from their disease. Two patients died without clinical or radiological evidence of disease progression, one from viral pneumonia and the other at home; post-mortem examination was not performed. Predicted median survival from the time of catheter insertion is 19 months (Figure 2).

### Discussion

The biochemical modulation of 5-FU with folinic acid has been an important recent advance in the systemic treatment of patients with widespread colorectal cancer (Kerr, 1989). In nine phase III studies which have compared systemic 5-FU with 5-FU and folinic acid the overall response rate for the combination is 23% compared with only 11% for 5-FU alone (Piedbois *et al.*, 1992). However, adding folinic acid also increases toxicity (Rustum, 1989). For example in one study in which high-dose folinic acid (500 mg $m^{-2}$ $week^{-1}$) was administered with 5-FU (0.6 g $m^{-2}$ $week^{-1}$), there was a 27% incidence of life-threatening diarrhoea (Petrelli *et al.*, 1987). Furthermore, increased response rates have not led to significantly improved survival (Piedbois *et al.*, 1992).

**Figure 1** Life table analysis of time to progression. Median survival is estimated at 8 months.

**Figure 2** Life table analysis of survival. Median survival is estimated at 19 months.

**Table III** Initial site of tumour progression in patients with progressive disease ($n$ = 24)

| | |
|---|---|
| Liver | 10 |
| Lung | 6 |
| Locoregional | 6 |
| Bone | 2 |
| Total | 24 |

For patients with metastases confined to the liver, targeting cytotoxics by regional administration is an attractive concept which may circumvent the restrictions of systemic chemotherapy. Based on a sound pharmacological rationale, this approach can produce high tumour response rates with minimal systemic toxicity. Three levels of targeting have been described (Widder *et al.*, 1979). At the first level selective delivery to the tumour-bearing organ is achieved. With second-level targeting, drug is directed to tumour rather than to normal tissue within the organ. Third-level targeting enhances uptake of drug by malignant cells. As hepatic metastases derive their blood supply predominantly from the hepatic artery (Breedis & Young, 1954), infusion of cytotoxics via the hepatic artery therefore achieves first- and, to a degree, second-level targeting. With this approach higher drug concentrations may be delivered to the tumour while overall systemic exposure, and hence toxicity, may be reduced. The reduction in systemic exposure is increased if drugs which have a high first-pass hepatic extraction are used.

FUDR, which has a first-pass extraction of 94–99%, is theoretically ideal for regional therapy (Ensminger *et al.*, 1978). For this reason, and also because its high solubility makes it suitable for use with totally implantable infusion pumps, FUDR has been accepted as the drug of choice for regional treatment of colorectal liver metastases. The response rates reported with IHA FUDR are high, ranging between 32% and 83% (Kemeny, 1992). Despite this, the results of seven randomised studies comparing it with systemic therapy are less encouraging. Only one of these, a French consortium study, has shown significantly improved survival with regional administration (Rougier *et al.*, 1992), but in this study half the patients in the control arm received no treatment at all. Furthermore, while systemic side-effects are not encountered with regional FUDR, hepatobiliary toxicity secondary to arterial infusion is a substantial problem. In the French study chemical hepatitis or biliary sclerosis occurred in 65% of patients.

Because systemic drug levels are negligible with IHA FUDR, poor survival is related to the rapid development of extrahepatic metastases (O'Connell, 1992). A logical approach, therefore, is to combine regional with adjuvant systemic treatment in order to inhibit extrahepatic progression. Safi *et al.* (1989) addressed this in a small randomised study which compared patients receiving combined IHA and systemic FUDR with a group who had IHA FUDR alone. Extrahepatic recurrence was 61% in the 23 patients treated with IHA FUDR alone compared with 33% in 21 patients who received combined therapy. Disappointingly, there was no difference in survival.

We have adopted an alternative approach. Because the first-pass hepatic extraction of 5-FU is between 19% and 54% (Ensminger *et al.*, 1978), intra-arterial administration of 5-FU is likely to produce not only high intrahepatic drug levels but also significant systemic levels as a result of unmetabolised drug spilling over from the liver into the systemic circulation. By combining this with folinic acid and using a prolonged infusional regimen, we aimed to maximise local tumour response and simultaneously delay extrahepatic progression. From our phase I study we found that the maximum safe dose of IHA 5-FU given over 24 h with FA 400 mg $m^{-2}$ was 1.5 g $m^{-2}$ $week^{-1}$. At 2.0 g $m^{-2}$ $week^{-1}$ grade 3 or 4 diarrhoea and/or vomiting occurred in half the patients. Although we have previously shown that there is significant regional advantage in giving FA via the hepatic artery, this was associated with arterial occlusion in some patients (Anderson *et al.*, 1992*b*). We therefore chose to administer FA systemically.

These results suggest that this approach is effective. The tumour response rate of 48% compares favourably with those of the previous HAI FUDR phase III studies for colorectal liver metastases (Kemeny, 1992). Systemic toxicity in this study was mild, with no grade 4 and only four cases of grade 3 toxicity. This is similar, in both spectrum and degree, to the toxicity encountered in studies of systemic 5-FU and folinic acid which utilised similar prolonged infusional schedules (De Gramont *et al.*, 1988; Johnson *et al.*, 1991), and therefore indicates that we were achieving equivalent therapeutic systemic drug levels.

Biliary sclerosis, commonly seen with regional FUDR infusion, was not seen, and only one patient had transient biochemical evidence of hepatitis. However, other local complications did occur. Despite prophylactic $H_2$-blockers, upper gastrointestinal haemorrhage occurred in three patients, presumably as a result of misperfusion. Although this should be minimised by careful ligation of any vessels arising from the hepatic artery which supply stomach or duodenum, it is recognised that collaterals may develop (Kemeny *et al.*, 1984). Early investigation of dyspeptic symptoms by endoscopy is therefore mandatory.

Hepatic abscess formation was another serious complication. In the patient infected with Gram-negative bacilli this was associated with chronic suppuration at the subcutaneous port site, while a hospital-acquired staphylococcal infection occurred in the other. Despite this, both patients responded to parenteral antibiotic therapy (although one required additional percutaneous drainage). One is still alive 21 months post catheterisation while the other died at 20 months of progressive liver disease.

The majority of patients who progressed did so at extrahepatic sites in the first instance, indicating that control of local disease was superior to the inhibitory effect of the systemic component. However, the median time to progression of 8 months suggests that disease progression was delayed. The high response rate and relative lack of toxicity suggest that this regimen warrants further evaluation. A phase III study comparing systemic and regional 5-FU and folinic acid regimens is now planned.

We gratefully acknowledge the support of BUPA Medical Foundation Ltd. and the European Community Concerted Action Programme.

### References

ANDERSON, J.H., KERR, D.J., COOKE, T.G. & MCARDLE, C.S. (1992*a*). A phase I study of regional 5-fluorouracil and systemic folinic acid for patients with colorectal liver metastases. *Br. J. Cancer*, **65**, 913-915.

ANDERSON, J.H., KERR, D.J., SETANOIANS, A., COOKE, T.G. & MCARDLE, C.S. (1992*b*). A pharmacokinetic comparison of intravenous versus intra-arterial folinic acid. *Br. J. Cancer*, **65**, 133-135.

BREEDIS, C. & YOUNG, G. (1954). The blood supply of neoplasms in the liver. *Am. J. Pathol.*, **30**, 969-985.

CADY, B. & STONE, M.D. (1991). The role of surgical resection of liver metastases in colorectal carcinoma. *Semin. Oncol.*, **18**, 399-406.

DE GRAMONT, A., KRULIK, M., CADY, J., LAGADEC, B., MAISANI, J.E., LOISEAU, J.P., GRANGE, J.D., GONZALEZ-CANALI, G., DEMUYNCK, B., LOUVET, C., SEROKA, J., DRAY, C. & DEBRAY, J. (1988). High-dose folinic acid and 5-fluorouracil bolus and continual infusion in advanced colorectal cancer. *Eur. J. Cancer Clin. Oncol.*, **24**, 1499-1503.

ENSMINGER, W.D., ROSOWSKY, A., RASO, V., LEVIN, D.C., GLUDE, M., COME, S., STEELE, G. & FREI, E. (1978). A clinical-pharmacological evaluation of hepatic arterial infusions of 5-fluorouracil. *Cancer Res.*, **38**, 3784-3792.

FORTNER, J.G., SILVA, J.S., COX, E.B., GOLBEY, R.B., GALLOWITZ, H. & MACLEAN, B.J. (1984). Multivariate analysis of a personal series of 247 patients with liver metastases from colorectal cancer. *Ann. Surg.*, **199**, 306-316.

GOLDBERG, J.A., LEIBERMAN, D.P. & MCARDLE, C.S. (1989). A useful maneuver for hepatic arterial catheterization in patients with metastatic hepatic disease and abnormal vascular anatomy. *Surg. Gynecol. Obstet.*, **169**, 71-72.

GOLDBERG, J.A., KERR, D.J., WATSON, D.G., WILMOTT, N., BATES, C.D., MCKILLOP, J.H. & MCARDLE, C.S. (1990*a*). The pharmacokinetics of 5-fluorouracil administered by arterial infusion in advanced colorectal hepatic metastases. *Br. J. Cancer*, **61**, 913-915.

JOHNSON, P.W.M., THOMPSON, P.I., SEYMOUR, M.T., DEASY, N.P., THURAISINGHAM, R.C., SLEVIN, M.L. & WRIGLEY, P.F.M. (1991). A less toxic regimen of 5-FU and high dose folinic acid for advanced gastrointestinal adenocarcinomas. *Br. J. Cancer*, **64**, 603-605.

KEMENY, N. (1992). Review of regional therapy of liver metastases in colorectal cancer. *Semin. Oncol.*, **2**, 155-162.

KEMENY, N. & SEITER, K. (1991). Therapy for colorectal cancer. *Curr. Opin. Oncol.*, **3**, 745-752.

KEMENY, N., DALY, J., ODERMAN, P., SHIKE, M., CHUN, H., PETRONI, G. & GELLER, N. (1984). Hepatic artery pump infusion: toxicity and results in patients with metastatic colorectal carcinoma. *J. Clin. Oncol.*, **2**, 595-600.

KEMENY, N., DALY, J., REICHMAN, B., GELLER, N., BOTET, J. & ODERMAN, P. (1987). Intrahepatic or systemic infusion of fluorodeoxyuridine in patients with liver metastases from colorectal carcinoma. *Ann. Intern. Med.*, **107**, 459-465.

KERR, D.J. (1989). 5-Fluorouracil and folinic acid: interesting biochemistry or effective treatment? *Br. J. Cancer*, **60**, 807-808.

LOKICH, J.J., AHLGREN, J.D., GULLO, J.J., PHILIPS, J.A. & FRYER, J.G. (1989). A prospective randomised comparison of continuous infusion fluorouracil with a conventional bolus schedule in metastatic colorectal carcinoma: a mid-Atlantic oncology program study. *J. Clin. Oncol.*, **7**, 425-432.

MILLER, A.B., HOOGSTRATEN, B., STAQUET, M. & WINKLER, A. (1981). Reporting results of cancer treatment. *Cancer*, **47**, 207-214.

O'CONNELL, M.J. (1992). Is hepatic infusion of chemotherapy effective treatment for liver metastases? No! *Important Adv. Oncol.*, 229-234.

PETRELLI, P., HERRERA, L., RUSTUM, Y., BURKE, P., CREAVEN, P., STULC, J., EMRICH, L.J. & MITTELMAN, A. (1987). A prospective randomised trial of 5-fluorouracil versus 5-fluorouracil and high dose Leucovorin versus 5-fluorouracil and methotrexate in previously untreated patients with advanced colorectal carcinoma. *J. Clin. Oncol.*, **5**, 1559-1565.

PIEDBOIS, P., BUYSE, M., RUSTUM, Y., MACHOVER, D., ERLICHMAN, C., CARLSON, R.W., VALONE, F., LABIANCA, R., DOROSHAW, J.H. & PETRELLI, N. (1992). Modulation of fluorouracil by Leucovorin in patients with advanced colorectal cancer: evidence in terms of response rate. *J. Clin. Oncol.*, **6**, 896-903.

PINEDO, H.M. (1988). Fluorouracil: biochemistry and pharmacology. *J. Clin. Oncol.*, **6**, 1653-1664.

ROUGIER, P., LAPLANCE, A., HUGUIER, M., HAY, J.M., OLLIVIER, J.M., ESCAT, J., SALMON, R., JULIEN, M., AUDY, J.R., GALLOT, D., GOUZI, J.L., PAILLER, J.L., ELISA, D., LACAINE, F., ROOS, S., ROTMAN, N., LUBOINSKI, M. & LASSER, P. (1992). Hepatic arterial infusion of floxuridine in patients with liver metastases from colorectal carcinoma: long term results of a prospective randomised trial. *J. Clin. Oncol.*, **7**, 1112-1118.

RUSTUM, Y.M. (1989). Toxicity and antitumor activity of 5-fluorouracil in combination with Leucovorin. *Cancer*, **63**, 1013-1017.

SAFI, F., BITTNER, R., ROSCHER, R., SCHUMACHER, K., GAUS, W. & BEGER, G.H. (1989). Regional chemotherapy for hepatic metastases of colorectal carcinoma (continuous intraarterial versus continuous intraarterial intravenous therapy). Results of a controlled clinical trial. *Cancer*, **64**, 379-387.

WATKINS, E., KHAZEI, A.M. & NAHRA, K.S. (1970). Surgical basis for arterial infusion chemotherapy of disseminated carcinoma of the liver. *Surg. Gynecol. Obstet.*, **130**, 581-605.

WIDDER, K.J., SENYEI, A.E. & RANNEY, D.F. (1979). Magnetically responsive microspheres and other carriers for the biophysical targeting of antitumour agents. *Adv. Pharmacol. Chemother.*, **16**, 213-271.

WOOD, C.B., GILLIS, C.R. & BLUMGART, L.H. (1976). A retrospective study of the natural history of patients with liver metastases from colorectal cancer. *Clin. Oncol.*, **2**, 285-288.